# The Effect of Back Massage on Blood Pressure in the Patients with Primary Hypertension in 2012-2013: A Randomized Clinical Trial

**Published:** 2014-10

**Authors:** Zinat Mohebbi, Mehdi Moghadasi, Kaynoosh Homayouni, Mohammad Hassan Nikou

**Affiliations:** 1 Department of Nursing, School of Nursing, Student Research Committee, Shiraz University of Medical Sciences, Shiraz, Iran;; 2 Department of Rehabilitation, School of Rehabilitation, Shiraz University of Medical Sciences, Shiraz, Iran;; 3 Department of Cardiology, School of Medicine cardiologist, Shiraz University of Medical Sciences, Shiraz, Iran

**Keywords:** Blood Pressure, Massage, Primary Hypertension

## Abstract

**Background: **Tension and stress are among the factors that lead to hypertension. In most individuals, behavioral strategies, such as relaxation and massage, are effective in controlling the individuals’ response to stress, thus reducing hypertension.

**Methods:** This non-blind clinical trial was conducted on 90 patients with primary hypertension. The patients were randomly divided into a control and an intervention group. In both groups, blood pressure was measured and recorded twice a week before and after a 10-min Swedish back massage and rest for 6 weeks. The study data were collected using a questionnaire including demographic information, a check list of blood pressure record, and a fixed manometer.

**Results:** In the intervention group, systolic and diastolic blood pressure decreased to 6.44 and 4.77 mmHg, respectively after back massage (P<0.001).

**Conclusion:** The obtained results were indicative of the effectiveness of back massage in reducing blood pressure in the study participants. Using stress control methods, such as massage, is a simple, acceptable, and teachable method for families to control blood pressure. After conducting more studies on this issue, back massage can be recommended as a non-pharmacological method to control blood pressure.

**Trial Registration Number**: IRCT2013112615552N1.

## Introduction

According to the report of the Seventh Joint National Committee on Prevention, Detection, Evaluation, and Treatment of High Blood Pressure (JNC 7), Blood Pressure (BP) is considered high if it exceeds 140/90 mmHg.^1^ Hypertension is in fact one of the main causes of the increase in cardiovascular diseases worldwide.^2^^,^^3^ About 50 million adults (18 years and above) in the United States suffer from hypertension.^4^ Hypertension is the most important risk factor for cardiovascular and chronic renal failure diseases, and imposes high costs on both individuals and societies. In the United States, for instance, more than 10 billion dollars are spent on this disorder every year.^5^ According to the statistics, one-fifth of the Iranian people (18.6%) over 15 years old suffer from this disease.^6^ Olney et al. suggested complementary medicine in order to reduce stress and control BP.^4^ In addition, Osborn et al. stated that use of complementary medicine could be effective in reducing BP, and this method was easy, available, and more cost-effective compared to medications.^7^ Considering the physiopathology of BP and the effective mechanism of massage therapy, relaxation through massage can facilitate the response of parasympathetic nerve, thus reducing heart rate, BP, and anxiety.^8^ During repeated sensory stimulation by massage, changes in neural cycles cause a variable activity in the autonomic nervous system, such as the BP regulation system.^9^ Systolic BP increases during acute stress, while diastolic BP increases only after long-term stress. Reduction of diastolic BP over time can be due to continuous sensory stimulation.^10^Aourell et al. suggested that repeated sensory stimulations during massage could result in changes in neural currents and automatic system activity and, consequently, could cause changes in BP and heart rate .^11^ In some studies, the sample size was small and in others, only women or men with hypertension were examined. In some other studies, the number of massage sessions was low. Hence, the present study aims to eliminate the gaps existing in the previous studies. We intended to study the effect of back massage, as a non-pharmacological method, on BP in the patients with primary hypertension. Therefore, this study can help the patients and their families to control this disease with minimum use of facilities and to prevent serious complications.

## Materials and Methods

This non-blind clinical trial was performed at Imam Reza Clinic affiliated to the Shiraz University of Medical Sciences, Shiraz, southern Iran from August to September 2013. The study was approved by the Ethics Committee of Shiraz University of Medical Sciences. In this research, after a heart specialist diagnosed primary hypertension, all the patients were sent to the researchers. The patients were selected based on the inclusion criteria of the study and then, the method of massage and use of oil during the massage were explained to them. Afterwards, the patients signed written informed consents and their BP was measured. Sampling lasted for 2 months and 17 days. The inclusion criteria of the study were not suffering from mental disorders, being between 30 and 70 years old, not participating in other relaxation programs, having no history of low BP (hypotension), having no skin or spine disorders, not using anti-stress drugs and painkillers, passage of at least 6 months from diagnosis of hypertension, using at least one type or at most three types of antihypertensive drugs, not changing the medications used during the intervention, systolic BP of 130–170 mmHg, diastolic BP of 80–120 mmHg, and not suffering from diabetes and other diseases, such as renal problems, tumor adrenal gland, and congenital heart defects, that increase BP. The patients who did not meet the inclusion criteria were not recruited into the study. The study sample size was determined based on the study by Hassanvand et al. entitled “The effect of back massage on BP and radial pulse of patients with primary hypertension”^12^ and using the following formula (d=10, S=8, α=0.05, β=0.2).

$$n=\frac{{\left({\text{Z}}_{1-\frac{\alpha }{2}}+{\text{Z}}_{1-\beta }\right)}^{2}\times 2{\text{S}}^{2}}{{\text{d}}^{2}}$$

$$n=\frac{\left(\text{1.96}+\text{0.84}\right)\text{22}\times \text{64}}{\text{100}}\;\;n=\text{40}$$

The samples were selected through convenience sampling. At first, the patients were checked for the inclusion criteria and the ones who were entered into the study filled out the demographic information questionnaire and the written informed consents. Overall, 80 patients were enrolled into the study and were randomly divided into an intervention and a control group each containing 40 patients. Additionally, five patients were added to each group because of the probability of loss; thus, each group consisted of 45 patients. In doing so, numbers 1–90 were assigned to the patients and 45 numbers were selected using the table of random numbers. Then, allocation of each 45-subject group to the intervention or the control group was determined by flipping a coin. 

**Figure 1:**
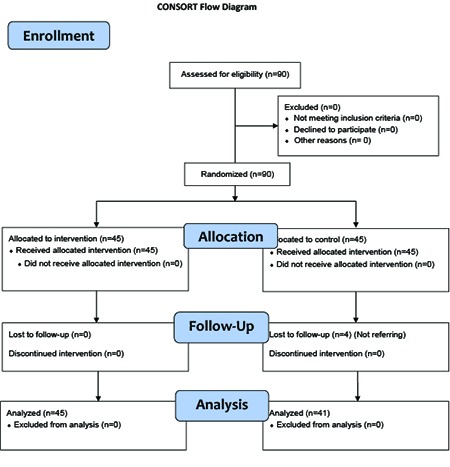
Flow diagram of the patients’ progress through the stages of the randomized clinical trial

In order to measure BP in this study, a digital manometer was used (Onyx model, Measurement accuracy=±3mmHg) whose reliability and validity have been confirmed previously. It was calibrated at the beginning and middle of the study and was used by the researcher who was unaware of the samples’ allocation to the study groups. Pearson correlation coefficients for reliability of systolic and diastolic BPs were obtained as 0.97 and 0.95, respectively. The protocol of the study has been presented in figure 1. In this study, back massage was performed by two graduate nursing students, one male and one female, who were trained by a physical medicine specialist. Afterward, the specialist checked their massage methods and confirmed that they were the same. The massage was Swedish massage from the neck to the back (from shoulders to waist) with compression (including a muscular pressure, one muscle or part of it, applying and then releasing pressure, proceeding to adjacent areas and repeating), continued with pushing, tapotement (tapping muscles with the side of the hand and fingers and side closed fist including more provocative movements), and then with wringing (two hands move against each other and the muscles are compressed between them). The intervention group referred on Saturdays and Sundays and the control group referred on Mondays and Tuesdays from 8 A.M. to 1 P.M. In both control and intervention groups, the patients rested for 5 min and then, their BPs were recorded. BP was recorded twice a week before every intervention for 6 weeks. In the intervention group, an oil (sesame oil) was rubbed with soft movements on the back of the patient and back massage was performed from the neck to the entire back area (from shoulders to waist) for 10 min. Immediately after the 10-min massage, BP was recorded again. In the control group, the patients rested for 10 min and BP was recorded afterwards. The study data were collected using a questionnaire including demographic information and a checklist of BP records. After data collection, they were entered into the SPSS statistical software (v. 15).The demographic data were analyzed using t-test and chi-square test. After calculating the means and standard deviations at different times, repeated measures ANOVA was used for analyzing the variables. P<0.05 was considered as statistically significant.

## Results

The intervention group included 25 females and 20 males, while the control group included 27 females and 18 males. However, four female participants in the control group were excluded from the study because they did not refer to the clinic; thus, the control group consisted of 41 patients. Comparison of demographic characteristics in the two groups has been presented in table 1. Accordingly, no significant difference was observed between the two groups regarding sex, education level, marital status, occupation, and age.

**Table 1:** The patients’ demographic data

**Variable**		**Intervention**	**Control**	**Total**	**Used test ** **and P value**
Age (Mean±SD)		56.80±8.16	59.17±8.5	57.98	t- test P=0.18
Sex	Male	20 (44.4%)	18 (40%)	38 (42.2%)	Chi-square P=0.67
	Female	25 (55.6%)	27 (60%)	52 (57.8%)	
Education level	Above diploma	7 (15.6%)	3 (6.7%)	10 (11.1%)	Chi-square P=0.48
	Diploma	13 (28.9%)	11 (24.4%)	24 (26.7%)	
	Below diploma	20 (44.4%)	24 (53.3%)	44 (48.9%)	
	Illiterate	5 (11.1%)	7 (15.6%)	12 (13.3%)	
Occupation	Employee	3 (6.7%)	1 (2.2%)	4 (4.4%)	Chi-square P=0.76
	Self-employed	5 (11.1%)	5 (11.1%)	10 (11.1%)	
	Retired	11 (24.4%)	13 (28.9%)	24 (26.7%)	
	Housewife	26 (57.8%)	26 (57.8%)	52 (57.8%)	
Marital status	Widowed	2 (%4.4)	4 (8.9%)	6 (6.7%)	Chi-square P=0.43
	Single	1 (2.2%)	0 (0)	1 (1.1%)	
	Married	42 (93.3%)	41 (%91.1)	83 (92.2%)	

**Figure 2:**
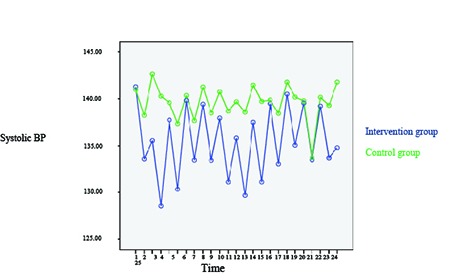
Trend of changes in the mean of systolic blood pressure in the intervention and control groups based on time

The means and standard deviations of changes in systolic BP were compared in the two groups before and after the intervention, and the results have been shown in table 2. As the table depicts, the mean of systolic BP decreased to 6.44 mmHg in the intervention group and the difference was statistically significant. In the control group also, the mean of systolic BP decreased to 2.31 mmHg, but the difference was not statistically significant. Comparison of the means of systolic BP in the intervention and control groups has been shown in figure 2.

**Table 2:** Comparison of changes in systolic blood pressure in the intervention and control groups before and after the intervention

**Sessions**	**Mean systolic blood pressure in the intervention group** **±** **SD**		**Mean systolic blood pressurein the control group ** **±** **SD**	
	**Before the intervention**	**After the intervention**	**Before the intervention**	**After the intervention**
1th session	141.28±14.18	133.55±13.88	140.97±14.44	138.21±12.47
2nd session	135.53±10.95	128.57±9.97	142.60±11.85	140.26±11.08
3rd session	137.73±11.77	130.33±10.70	139.58±11.41	137.34±9.88
4th session	139.82±10.64	133.44±10.77	140.34±10.74	137.65±9.54
5th session	139.42±9.07	133.42±8.22	141.26±9.79	138.46±8.58
6th session	137.93±9.67	131.11±9.14	140.73±9.63	138.68±8.43
7th session	135.80±9.19	129.66±9.02	139.68±9	138.56±7.42
8th session	137.48±7.54	131.11±8.14	141.46±7.89	139.70±6.75
9th session	139.48±8.74	133.04±8.20	139.85±8.59	138.43±7.85
10th session	140.48±8.01	135.04±7.95	141.78±8.55	140.17±7.56
11th session	139.55±7.48	133.46±7.86	139.78±7.84	133.68±8.14
12th session	139.22±7.22	133.66±8.24	140.14±7.49	139.29±7.08
Used test	Repeated measures ANOVA		Repeated measures ANOVA	
F	11.39		2.29	
P value	P<0.001		P=0.052	

**Figure 3:**
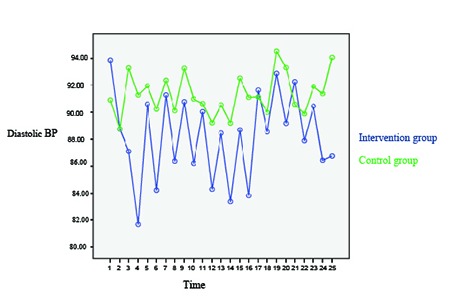
Trend of changes in the mean of diastolic blood pressure in the intervention and control groups based on time

Comparison of changes in the means and standard deviations of diastolic BP in the intervention and control groups before and after the intervention has been presented in table 3. Based on the table, the mean of diastolic BP decreased to 4.77 mmHg in the intervention group and the difference was statistically significant. In addition, the mean of diastolic BP decreased to 1.5 mmHg in the control group, but the difference was not statistically significant. Comparison of the means of diastolic BP in the intervention and control groups has been shown in figure 3.

**Table 3:** Comparison of the mean changes in diastolic blood pressure in the intervention and control groups before and after the intervention

**Sessions**	**Mean diastolic blood pressure in the intervention group** **±** **SD**		**Mean diastolic blood pressure in the control group** **±** **SD**	
	**Before the intervention**	**After the intervention**	**Before the intervention**	**After the intervention**
1th session	93.86±14.43	88.73±12.64	90.90±16.19	88.75±14.68
2nd session	87.08±10.33	81.66±8.91	93.26±15.40	91.26±14.13
3rd session	90.60±11.86	84.22±11.13	91.95±10.57	90.21±9.26
4th session	91.26±11.01	86.35±10.12	92.36±11.66	90.09±10.38
5th session	90.77±11.67	86.20±10.33	93.24±11.33	90.97±10.85
6th session	90.02±10.53	84.28±8.03	90.63±9.35	89.21±8.07
7th session	88.44±11.54	83.35±9.76	90.53±8.45	89.19±7.77
8th session	88.68±9.46	83.80±8.99	92.53±10.78	91.09±9.39
9th session	91.62±11.04	88.53±9.90	91.12±10.46	89.97±8.85
10th session	92.86±9.35	89.17±8.75	94.51±10.15	93.29±9.31
11th session	92.26±8.49	87.88±7.69	90.58±7.36	89.89±7.02
12th session	90.44±7.97	86.42±6.89	91.90±9.64	91.36±8.75
Used test	Repeated measures ANOVA		Repeated measures ANOVA	
F	7.36		1.2	
P value	P<0.001		P=0.23	

The changes in systolic and diastolic BP were compared between the intervention and the control group from sessions 1–12, and the results of repeated measures ANOVA indicated that the differences were statistically significant (P<0.001). Furthermore, the effect of time, group, and time*group on systolic and diastolic BP in the two groups was assessed using repeated measures ANOVA, and the results have been depicted in table 4. According to the heart specialist comments, decrease in systolic and diastolic BP was clinically significant only in the intervention group.

**Table 4:** The effect of time, group, and time*group on systolic and diastolic blood pressure

**Effect**	**Blood pressure**	**df**	**F**	**P value**
Time	Systolic	24	8.9	P<0.001
	Diastolic	24	4.81	P<0.001
Group	Systolic	1	10.77	P=0.002
	Diastolic	1	5.34	P=0.02
Time*Group	Systolic	24	3.96	P<0.001
	Diastolic	24	2.77	P<0.001

## Discussion

The results of the present research showed that after the intervention, the patients’ means of diastolic and systolic BP respectively decreased to 6.44 and 4.77 mmHg in the intervention group and to 2.31 and 1.51 mmHg in the control group. Thus, the results indicated the effectiveness of massage in controlling BP. In the same line, a study in Hong Kong showed that massage by superficial stroke method for 10 min performed for 7 consecutive days was effective in reducing hypertension in the elderly individuals with stroke.^13^ A similar study also investigated the effects of Swedish massage on BP, heart rate, and inflammatory markers in the women with hypertension. In that study, the participants were divided into an intervention and a control group each containing eight patients. The results revealed a significant reduction in systolic and diastolic BP after the fourth session.^14^Consistently, another study showed that systolic and diastolic BPs decreased after 10 sessions of back massage. In that study, however, the study participants served both as the intervention and the control group.^12^One other study in Karolina also evaluated the effect of massage by superficial stroke method on mental and physical symptoms of rehabilitation patients. The findings of that study demonstrated a significant reduction in BP after the intervention.^15^ However, another study on massage showed no significant changes in systolic and diastolic BP.^16^ Nonetheless, the results of the studies by Christin M. Olney, Mohammad Reza Yeganehkhah et al., Moeini et al., ŞebnemÇinar et al., and MakNamara et al. showed that massage therapy considerably decreased diastolic and systolic BP.^4^^,^^17^^-^^20^ This is indicative of the effect of this non-pharmacological treatment method on reducing BP in the elderly individuals with hypertension.

One of the limitations of the current study was that back massage was performed on the aforementioned areas for 10 min. Therefore, further studies are recommended to apply massages on the whole body and for a longer period of time. Yet, number of massage sessions and number of participants were the strong points of this study. 

## Conclusion

Based on the results of the present study, 12 sessions of back massage reduced systolic and diastolic BP in the patients with hypertension. Thus, after conducting more studies on this issue, back massage can be recommended as a non-pharmacological method for BP control. 
